# Ceramides are early responders in metabolic syndrome development in rhesus monkeys

**DOI:** 10.1038/s41598-022-14083-3

**Published:** 2022-06-15

**Authors:** Alex B. Smith, Jonah P. Schill, Ruth Gordillo, Grace E. Gustafson, Timothy W. Rhoads, Maggie S. Burhans, Aimee T. Broman, Ricki J. Colman, Philipp E. Scherer, Rozalyn M. Anderson

**Affiliations:** 1 Department of Medicine, University of Wisconsin-Madison, Madison, WI USA; 2 Department of Internal Medicine, Touchstone Diabetes Center, The University of Texas Southwestern Medical Center, Dallas, TX USA; 3 Biostatistics and Medical Informatics, University of Wisconsin-Madison, Madison, WI USA; 4 Wisconsin National Primate Research Center, University of Wisconsin-Madison, Madison, WI USA; 5 Department of Cell and Regenerative Biology, University of Wisconsin-Madison, Madison, WI USA; 6 Department of Cell Biology, The University of Texas Southwestern Medical Center, Dallas, TX USA; 7 Geriatric Research, Education, and Clinical Center, William S. Middleton Memorial Veterans Hospital, Madison, WI USA

**Keywords:** Obesity, Metabolic diseases, Biomarkers, Endocrinology

## Abstract

Metabolic syndrome increases risk of complicating co-morbidities. Current clinical indicators reflect established metabolic impairment, preventing earlier intervention strategies. Here we show that circulating sphingolipids are altered in the very early stages of insulin resistance development. The study involved 16 paired overweight but healthy monkeys, one-half of which spontaneously developed metabolic syndrome over the course of 2 years. Importantly, animals did not differ in adiposity and were euglycemic throughout the study period. Using mass spectrometry, circulating sphingolipids, including ceramides and sphingomyelins, were detected and quantified for healthy and impaired animals at both time points. At time of diagnosis, several ceramides were significantly different between healthy and impaired animals. Correlation analysis revealed differences in the interactions among ceramides in impaired animals at diagnosis and pre-diagnosis when animals were clinically indistinguishable from controls. Furthermore, correlations between ceramides and early-stage markers of insulin resistance, diacylglycerols and non-esterified fatty acids, were distinct for healthy and impaired states. Regression analysis identifies coordinated changes in lipid handling across lipid classes as animals progress from healthy to insulin resistant. Correlations between ceramides and the adipose-derived adipokine adiponectin were apparent in healthy animals but not in the metabolically impaired animals, even in advance of loss in insulin sensitivity. These data suggest that circulating ceramides are clinically relevant in identifying disease risk independent of differences in adiposity, and may be important in devising preventative strategies.

## Introduction

Metabolic syndrome is associated with an increased risk for a variety of diseases, and it negatively impacts quality of life of millions of people in the US alone every year^1^. Current diagnosis of risk for metabolic impairment hinge on broad assessments such as BMI, abdominal circumference, and family history^2^. Though inherently linked to adiposity, not all persons with obesity suffer from metabolic syndrome. According to the CDC 2016 reports, obesity affects about 40% of the adult population in the United States, and while 85% of the nation’s 30 million diabetic adults are overweight or obese, about 15% of the obese population is diabetic. This indicates that adiposity alone cannot explain metabolic dysfunction. The endocrine nature of adipose tissue has been offered as a possible explanation for the inconsistency between adiposity and metabolic outcomes in preclinical trials, where disease may be a result of disrupted adipose function not adiposity per se^3,4^. Current methods for diagnosing diabetes and pre-diabetes are based on fasting plasma glucose levels or glycated hemoglobin, an indicator of blood glucose levels over recent months. While these diagnoses are widely used, rather than being predictive, they serve as a verification of established metabolic dysfunction^5^. A method of specifically screening for the future onset of metabolic syndrome prior to dysregulated glycemia and independent of adiposity is not currently available.

Due to well established anatomical, physiological, and behavioral similarities between rhesus monkeys (*Macaca mulatta*) and humans, health-related biomarkers identified in this model are likely translatable to humans^5^. Rhesus monkeys share many of the same diseases and disorders as humans, including metabolic dysfunction, and cross-sectional studies show that lipoprotein profiles and plasma triaclyglycerol (TAG) levels are valuable diagnostic indicators of metabolic dysfunction in the same manner for both humans and monkeys^6,7^. In our previous study of metabolic health in rhesus monkeys, several species of lipids were significantly impacted by progression to insulin resistance^8^. Quantitative analysis of circulating lipids revealed elevated circulating levels of triacylglycerols in the insulin resistant monkeys at time of diagnosis, that along with adiposity and insulin resistance met the criteria for metabolic syndrome. Changes in chain length and saturation of discrete diacylglycerols (DAG) formed the basis of a predictive model that could identify insulin resistance in advance of impaired fasting glucose (IFG). Correlation analysis showed substantial differences in interactions among lipid species in insulin resistant animals compared to healthy controls, pointing to differences in lipid handling, including elongation and desaturation, early in the development of insulin resistance. The adipose-derived peptide hormone adiponectin also featured in a predictive model of insulin resistance onset^8^. Adiponectin has been linked to insulin sensitivity and regulation of inflammatory signaling^9^, although its connection to changes in composition of circulating lipids has not been established.

Sphingolipids, including ceramides, are among the circulating lipids that have previously been implicated in insulin resistance and diabetes in humans^10–12^ and in monkeys^13^, with broader links to cardiovascular disease in human studies^14,15^. Although the tissue source of circulating ceramides is not clear, within cells the de novo synthesis of ceramides occurs in the endoplasmic reticulum (ER) via condensation of palmitoyl-CoA and serine by the enzyme serine palmitoyl-transferase with subsequent reduction to produce sphinganine (dihydrosphingosine) (Fig.S1). Acylation of sphinganine by different ceramide synthases creates a variety of dihydroceramides, which can then be desaturated by dihydroceramide desaturase, creating the diverse ceramide pool^16^. Alternatively, ceramides can be synthesized by the salvage pathway from sphingosine. Further synthetic reactions involve ceramides as precursors to glycosphingolipids and sphingomyelins, the latter of which are quite abundant in circulation. The current study quantifies plasma sphingolipid species from these various classes to determine whether there are differences early in the development of insulin resistance, prior to loss of euglycemia, and if sphingolipid changes correlate with other insulin responsive biomolecules such DAG. Adiposity was equivalent between healthy and impaired animals, affording the possibility for new insight into the relationships among circulating lipids and the adipose-derived insulin sensitizer adiponectin independent of differences in adiposity.

## Materials and methods

### Animal care and assessments

Animals were maintained in accordance to guidelines for the ethical care and treatment of animals as approved by the Institutional Animal Care and Use Committee of the Graduate School of the University of Wisconsin-Madison. This study design has been previously reported^8^. Briefly, 16 adult male rhesus monkeys of Indian origin from 10 to 22 years of age were housed individually at the Wisconsin National Primate Research Center. Rooms were maintained at 21–26 °C with ~ 50–65% relative humidity. Animals were allowed ad libitum access to food for 6–8 h per day and were fed a pelleted, semipurified diet (15% lactalbumin, 10% corn oil, and ~ 65% carbohydrate in the form of sucrose and cornstarch). Animals had continuous access to water and were monitored daily. Assessments have been previously described^17^ and included: body weight (weekly); body composition (every 6 months via dual energy X-ray absorptiometry); glucoregulatory function (every 6 months via levels of fasting plasma glucose and insulin, and insulin sensitivity measured using a frequently sampled intravenous glucose tolerance test (FSIGT). Plasma samples used for lipidomic analysis were drawn > 3 h following glucose infusion during the FSIGT, a time point when baseline measures of insulin and glucose are reestablished. Plasma samples were stored at − 80 °C prior to analysis.

### Ceramide and sphingolipid quantitation

Plasma sphingolipids were isolated from plasma by performing a two-phase liquid–liquid extraction utilizing 15:85 (v/v) isopropanol:ethyl acetate. Sphingolipids were quantified by liquid chromatography-electrospray ionization tandem mass spectrometry with a Shimadzu LCMS-8050 Triple Stage Quadrupole Mass Spectrometer (Shimadzu Scientific Instruments, Columbia, MD) equipped with a DUIS (dual ionization source) operating in electrospray mode and interfaced with a Shimadzu Nexera X2 UHPLC system (Shimadzu Scientific Instruments) as previously described^18^. Briefly, lipids were separated on a 2.1 (i.d.) × 150 mm Kinetex C8, 2.6-micron core–shell particle column (Phenomenex, Torrance, California, USA). Sphingolipid species were identified based on exact mass and fragmentation patterns and verified by sphingolipids true standards. Concentrations were determined according to calibration curves using peak-area ratio against a corresponding internal standard (Cer/Sph Mixture II, Avanti Polar lipids, Alabaster, AL). Data are expressed as nanograms of metabolite per mL of plasma. LabSolutions V 5.82 and LabSolutions Insight V 2.0 program packages were used for data processing (Shimadzu Scientific Instruments).

### Fatty acid composition analysis

As previously described^8^, lipid species were extracted from fasting plasma^19^, then separated by silica gel thin layer chromatography using petroleum ether-diethyl ether-acetic acid (80:30:1) as the developing solvent. Nonesterified fatty acids (NEFA), and diacylglycerols (DAG) were scraped, methylated, and analyzed by gas–liquid chromatography and identified by comparison of retention times with authentic standards (Sigma). Internal standards included pentadecanoic acid (C15:0) to control for transmethylation efficiency, and heptadecanoic acid (C17:0) to allow for calculation of absolute concentrations.

### Statistics

All analyses were conducted both cross-sectionally and as two-year transitions. Variables measured at 2 years before diagnosis, at time of diagnosis, and the difference calculated for each individual animal between the two time points, each analyzed separately. For each individual species, the odds ratio (OR), confidence intervals, and unadjusted p values were calculated using impairment status (insulin sensitive or insulin resistant) as the binary outcome in logistic regression. We report odds ratios that summarize the change in odds of impairment for a one standard deviation change in the variable. Relationships between measured variables were summarized using non-parametric rank-based correlations (Spearman’s). Where indicated, correlations that were in absolute value greater than 0.7 are reported (Figs. 2 and 3). For analysis of variance in the individual species of sphingomyelins the Brown-Forsythe method was between groups of healthy and impaired at each time point, and the Pittman-Morgan test was used for within group between draw comparisons. Effect size plots show measures across groups and differences in group means with effect size shown as a 95% graded sampling distribution. Construction of coefficient of variation heatmap and estimation plot analysis via bootstrap resampling was performed in Rstudio (v. 1.4.1717; R version 4.1.1) using the gplots (v. 3.1.1) and dabestr (v. 0.3.0)^20^ packages. Five thousand re-samplings were performed to construct the 95% confidence interval distribution around the mean difference between healthy and impaired groups.

## Results

### Circulating lipids are early responders in development of insulin resistance

**Figure 1:**
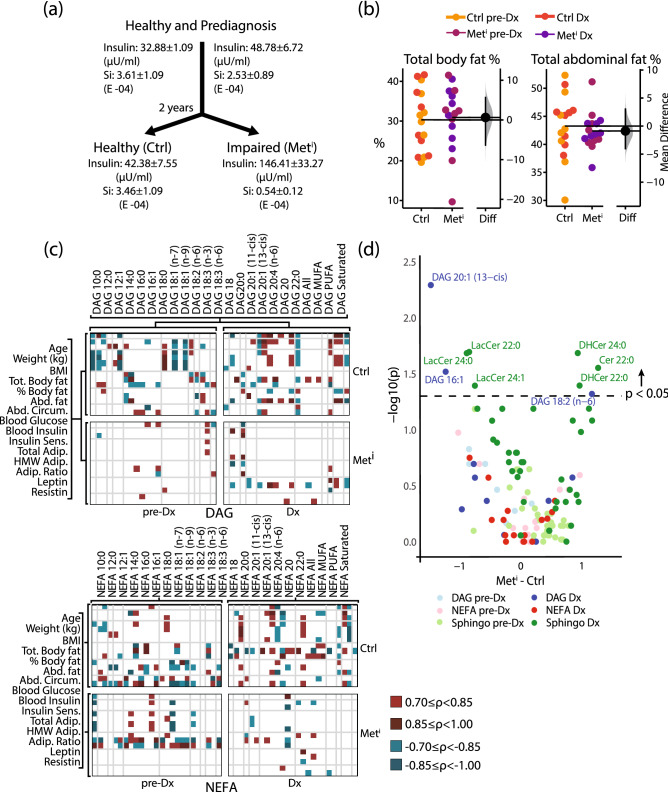
Changes in lipid metabolism with insulin resistance are independent of adiposity. (**a**) Schematic of study design, showing spontaneous development of hyperglycemia and loss of insulin sensitivity in half of the cohort. (**b**) Estimation plot displaying percent body fat and percent abdominal fat pre-diagnosis (pre-Dx) and at diagnosis (Dx) for healthy control (Ctrl) and metabolically impaired (Met^i^) animals. (**c**) Correlation between listed variables in control (Ctrl) and impaired (Met^i^) animals at diagnosis of insulin resistance (Dx) and 2 years prior (pre-Dx), DAG: diacylglycerols, NEFA: non-esterified fatty acids. (**d**) Volcano plot displaying difference in scored means of detected lipids between control (Ctrl) and impaired (Met^i^) groups at pre-diagnosis (pre-Dx; light dots) and at diagnosis (Dx; dark dots) time points (color-coded as indicated) against the log^10^ of the univariate p values (unadjusted). Volcano plot means for each species are normalized to sample standard deviation then means between impaired and healthy groups are compared. For all data, n = 8 animals per group per time point.

This study was designed to identify factors responding to insulin resistance development independent of age, body weight, and adiposity, and in advance of changes in glycemic status (Fig. 1a). Middle-aged male rhesus monkeys (age range 10–22 years; n = 8) were identified as insulin resistant when the following criteria were satisfied: fasting insulin > 70 U/ml and insulin sensitivity index (Si < 2 (E−04)) as determined by frequently sampled intravenous glucose tolerance test (FSIGT) in combination with an irregular glucose response curve. Control animals (n = 8) were pair-matched by age and weight at diagnosis. Plasma samples taken at time of diagnosis were analyzed and compared to samples from the same monkeys taken two years prior at which point all 16 were insulin sensitive. Both groups of monkeys did not differ at either timepoint for total body fat percent fat, or abdominal fat as determined by Dual X-ray absorptiometry (Fig. 1b), and both groups were euglycemic at both time points despite differences in insulin sensitivity. Key findings from our original investigation included changes in diacylglycerols (DAG) and nonesterified fatty acids (NEFA) composition that were associated with onset of insulin resistance, and differences in correlations among lipids in circulation, in particular for DAG and NEFA, that were evident either before (DAG only) or at the time of diagnosis (both DAG and NEFA). Here, correlation analysis was conducted to determine whether insulin sensitivity might impact the relationship between lipids and a selection of biometric and metabolic health indices, including body weight, adiposity, glucoregulatory parameters, and circulating adipokines. Correlations were plotted and color coded, where strong (magnitude R > 0.7) positive correlations were shaded red, and strong negative correlations were shaded blue, with darker shading indicating a stronger correlation (Fig. 1c). Strong correlations were detected for both DAG and NEFA from healthy animals at both time points, suggesting an underlying relationship between these circulating lipids and the biometric and metabolic health indices. For DAG, correlation patterns were notably different between control and insulin resistant animals at time of diagnosis and at two years prior to diagnosis when the monkeys that would go on to become insulin resistant were as yet clinically indistinguishable from healthy controls. For NEFA, the number of strong correlations were diminished only at the time of diagnosis of insulin resistance, but not before. These data show that correlations between lipids and biometric and health indices are lipid class specific and not uniform in their sensitivity to insulin resistance development and onset. Sphingolipid species were extracted from plasma and identified via liquid chromatography/electrospray ionization/tandem mass spectrometry. For each individual species the OR, confidence intervals, and unadjusted p values were calculated using the Wilcoxon test where insulin sensitivity (healthy or impaired) served as the binary outcome (Table S1). To get an overview of circulating lipids as a function of insulin sensitivity status, all species detected within DAG, NEFA, and sphingolipid classes were compared for impaired animals versus controls at time of diagnosis and at the earlier time point when all of the animals were metabolically healthy. Plotting the log of the univariate significance against the group mean differences normalized to units of standard deviation (Fig. 1d), several features were identified. Among these are DAG species that formed the basis of a predictive model for insulin resistance^8^, in addition to several ceramide precursors and derivatives. These data support the idea that circulating lipid composition changes with insulin sensitivity status.

### Changes in circulating ceramides mark onset of insulin resistance

**Figure 2:**
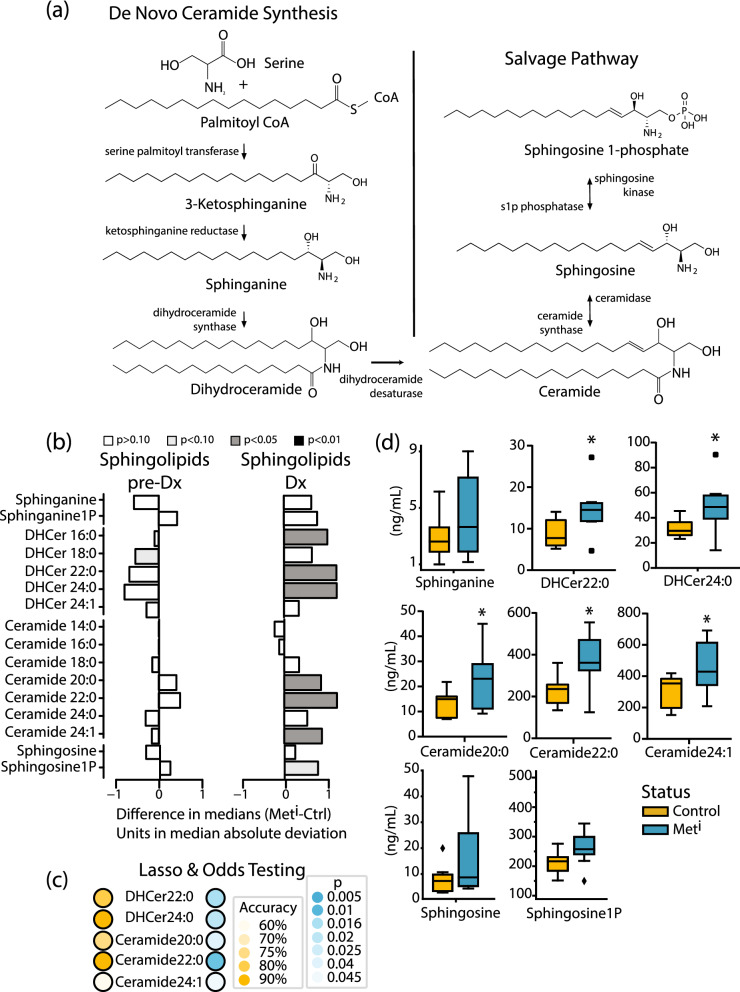
Discrete ceramide species mark the onset of insulin resistance. (**a**) Schematic showing synthetic pathways for production of detected sphingolipid species via de novo synthesis and the salvage pathway. (**b**) Differences in abundance of circulating ceramide species and precursors between control (Ctrl) and metabolically impaired (Met^i^) animals pre-diagnosis (pre-Dx) and at time of diagnosis (Dx). Data are shown as the difference in medians divided by the median absolute deviation for each species. Statistical significance based on univariate analysis (two-sample Wilcoxon) is shaded as indicated (unadjusted p values). (**c**) Lasso logistic regression showing ability of sphingolipid species to predict insulin resistance, with significance of regression based predictive p values and accuracy indicated by color gradient. (**d**) Abundance of circulating ceramide species (nanograms/mL) in control (Ctrl) and metabolically impaired (Met^i^) animals at diagnosis. DHCer: dihydroceramide, *p values < 0.05 (unadjusted). For all data, n = 8 animals per group per time point.

In the ceramide de novo synthesis pathway (Fig. 2a), broad scale changes in abundance of lipids were detected in metabolically impaired animals at time of diagnosis. In the feather plots shown, data are displayed as the difference in medians adjusted to unit deviation between impaired and healthy animals at diagnosis, and between pre-impaired and healthy at the prior time point (Fig. 2b). The OR, confidence intervals, and p values were calculated using insulin sensitivity status as the binary outcome (Table S1) (Fig. 2c). Sphinganine is formed in an early step in de novo synthesis and is a precursor for the dihydroceramides. The phosphorylated form of sphinganine is not part of the synthetic pathway but has been implicated in the TGF-beta/Smad2/3 signaling pathway^21^. Neither sphingoid base was significantly different between healthy and impaired at the time of diagnosis of insulin resistance, although values were numerically higher in the impaired. Twenty-five ceramide species were identified including 5 species of dihydroceramides and 7 species of ceramides. Of the dihydroceramides, dihydroceramide 22:0 and dihydroceramide 24:0 were significantly increased in metabolically impaired animals at time of diagnosis (Fig. 2d). Among the ceramide species, ceramide 20:0, ceramide 22:0, and ceramide 24:1 showed modest but significantly higher levels in plasma from impaired animals compared to healthy animals (Fig. 2d). None of the species of ceramides differed in abundance at the earlier time point when all animals were clinically healthy. Sphingosine-1 phosphate and sphingosine, precursors in the ceramide salvage pathway, were not significantly different between healthy and impaired although values were numerically higher at the time of diagnosis of insulin resistance.

### Complex glycosphingolipids and sphingomyelins are sensitive to onset of insulin resistance

**Figure 3:**
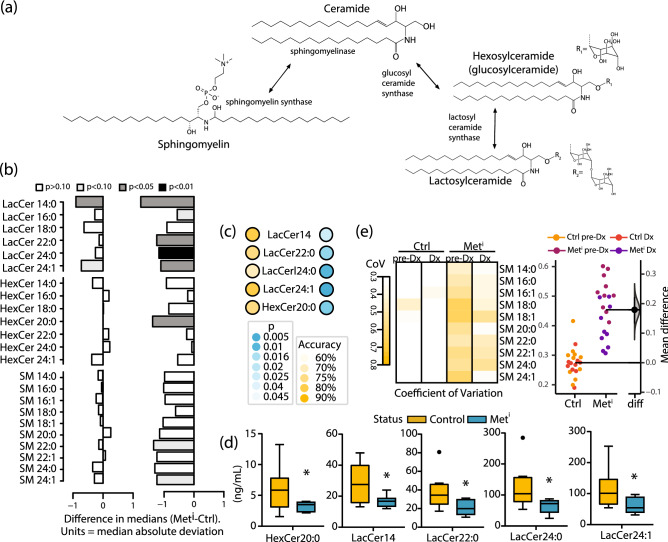
Sphingomyelins respond to insulin resistance. (**a**) Schematic showing downstream ceramide derivatives sphingomyelins and glycosphingolipids, including hexosylceramides and lactosylceramides. (**b**) Differences in circulating abundance of hexosylceramides (HexCer) and lactosylceramides (LacCer) and sphingomyelins (SM) between control (Ctrl) and metabolically impaired (Met^i^) animals pre-diagnosis (pre-Dx) and at time of diagnosis (Dx). Data are shown as the difference in medians divided by the median absolute deviation for each species. Statistical significance based on univariate analysis (two-sample Wilcoxon) is shaded as indicated (unadjusted p values). (**c**) Lasso logistic regression showing ability of indicated sphingolipids to predict insulin resistance, with significance of regression based predictive p values and accuracy indicated by color gradient. (**d**) Abundance of circulating hexosylceramide (HexCer) and lactosylceramide (LacCer) species (ng/mL) in control (Ctrl) and metabolically impaired (Met^i^) animals at time of diagnosis, *p values < 0.05 (unadjusted). (**e**) Left: heatmap of coefficient of variation (population standard deviation/population mean) of individual sphingomyelin species shown for control (Ctrl) and metabolically impaired (Met^i^) animals pre-diagnosis (pre-Dx) and at time of diagnosis (Dx). Right: bootstrapped mean difference 95% confidence intervals for sphingomyelins from control (Ctrl) and metabolically impaired (Met^i^) animals pre-diagnosis (pre-Dx) and at time of diagnosis (Dx) (color coded as indicated). For all data, n = 8 animals per group per time point.

Complex glycosphingolipids, including hexosylceramides and lactosylceramides, are derived from ceramides, as are sphingomyelins (Fig. 3a). Mass spectrometry identified circulating complex glycosphingolipids, including 7 species of hexosylceramides and 6 species of lactosylceramides. In the feather plots shown, data are displayed as the difference in medians adjusted to unit deviation between impaired and healthy animals at diagnosis, and between pre-impaired and healthy at the prior time point (Fig. 3b). As before, for each individual species the OR, confidence intervals, and p values were calculated using insulin sensitivity status as the binary outcome (Table S1) (Fig. 3c). Of these, hexosylceramide 20 was significantly lower in the impaired than for controls. Lactosylceramides as a group were generally of lower abundance in the impaired animals, although only individual species of lactosylceramides 14, 22, 24, and 24:1 were significantly different (Fig. 3d). In addition to the above complex ceramides, 10 species of sphingomyelins were also identified. As a group the sphingomyelins were lower in abundance in the metabolically impaired animals although none reached statistical significance. Unexpectedly, a pattern of increased variance was detected across the sphingomyelin class for metabolically impaired monkeys (Fig. 3e), and this heterogeneity was even evident two years before diagnosis, when the monkeys that would go on to become insulin resistant were clinically indistinguishable from healthy controls.

### Sphingolipid homeostasis is altered in advance of metabolic syndrome onset

**Figure 4:**
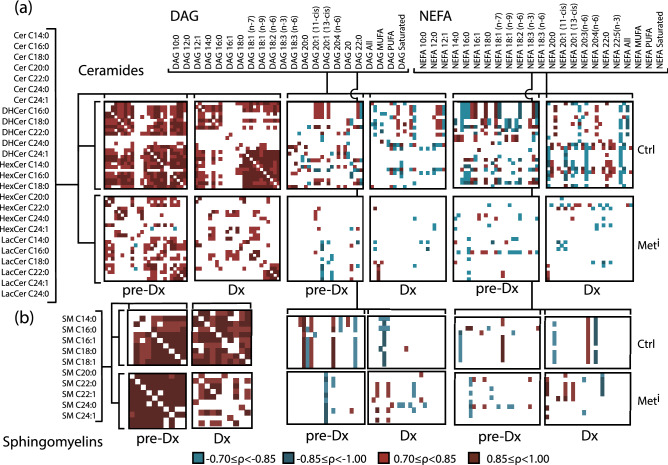
Interactions among sphingolipid species and other circulating lipids. Correlations were calculated separately for control (Ctrl) and metabolically impaired (Met^i^) animals pre-diagnosis (pre-Dx) when all animals were classified as healthy and at time of diagnosis (Dx). Strong positive or negative correlations ( >|0.7|) are indicated by color according to the legend, blank spaces indicate correlations that fall below threshold ( <|0.7|) or species that fall below detection. (**a**) Left: ceramides against ceramides, middle: ceramides against diacylglycerols (DAG), right: ceramides against nonesterified fatty acids (NEFA). (**b**) Left: sphingomyelins against sphingomyelins (SM), middle: sphingomyelins against diacylglycerols (DAG), right: sphingomyelins against nonesterified fatty acids (NEFA). (DHCer: Dihydroceramide, HexCer: Hexosylceramide, LacCer: Lactosylceramide) n = 8 animals per group per time point.

Previous work had pointed to differences in correlations among DAG and NEFA in metabolically impaired animals compared to healthy animals^8^; however, the full extent of lipid metabolic adaptation has not been fully captured. In particular, it is not known whether changes in associations among sphingolipid classes are also observed as a function of insulin sensitivity status, or if the insulin sensitivity in associations among adipose tissue derived lipids might also extend to sphingolipids, whose tissue of origin is not yet known. Spearman rank correlations were calculated for each species at each time point for healthy and impaired animals. Correlations were plotted and color coded as above with strong (magnitude R > 0.7) positive correlations shaded red, and strong negative correlations shaded blue. In healthy animals, associations among ceramide species were exclusively positive with ceramides and lactosylceramides in particular correlating strongly within and among pathway derivatives. A clear difference in the correlation matrix was observed at the time of diagnosis of metabolic syndrome, with many of the positive associations diminished, and a suggestion of forthcoming metabolic impairment was evident even at the earlier time point when impaired and healthy animals were clinically indistinguishable (Fig. 4a). Associations among lipid species also differed between healthy and impaired when ceramides were plotted against DAG and NEFA, lipid classes that we previously identified in the early response to metabolic syndrome. Interactions among sphingomyelins were calculated, and here too correlations were positive among species for the healthy animals and in the impaired animals. These strong correlations were unexpected given the increased heterogeneity in species abundance in the impaired animals. In contrast to the ceramides, only a few strong correlations dropped out at time of diagnosis for sphingomyelins. (Fig. 4b). Furthermore, the sphingomyelins did not show strong correlations against DAG and NEFA and the correlation matrices were similar for healthy and impaired animals.

### Broad-scale changes in sphingolipid interactions are coincident with development of insulin resistance

**Figure 5:**
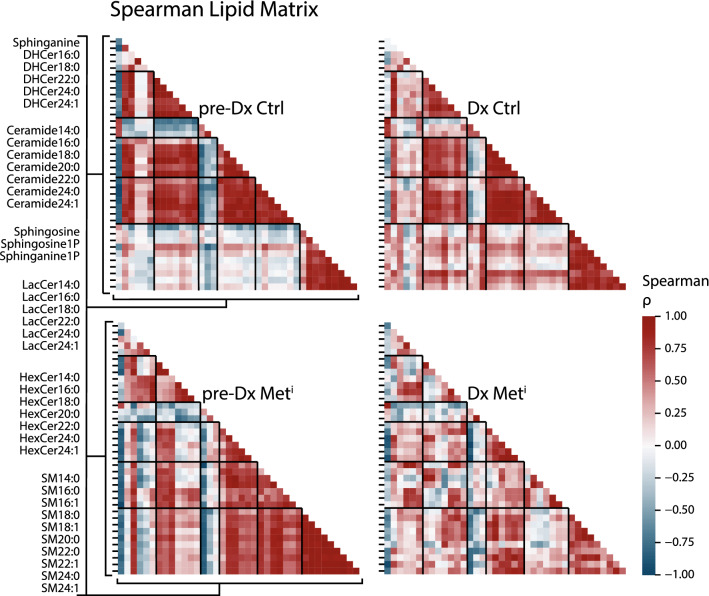
Correlation patterns among circulating sphingolipids during development of insulin resistance. Spearman’s rank correlations were calculated for individual lipids separately for control (Ctrl) and metabolically impaired (Met^i^) animals pre-diagnosis (pre-Dx) when all animals were classified as healthy and at time of diagnosis (Dx). Correlation coefficients colored according to the legend, showing positive and negative correlations. Each triangle is a correlation matrix of all paired lipid species for which the order of species is retained as shown on the left, where the blocks indicated on the illustration correspond to the groups of species listed. Species include dihydroceramide (DHCer), Ceramide (Cer), hexosylceramides (HexCer) and lactosylceramides (LacCer) and sphingomyelins (SM), n = 8 per group per time point.

To gain insight into the sphingolipid response to development and onset of insulin resistance, larger more inclusive correlation matrices were generated using all detected sphingolipids. For a holistic view, regression cut-offs were not applied to these matrices, but instead all associations are depicted to create correlation patterns. For healthy animals the correlation matrix was remarkably stable between draws, notwithstanding the two-year gap between measures (Fig. 5). A striking difference in correlations among lipid species was detected in the metabolically impaired animals, both at time of diagnosis and two years prior when animals were as yet insulin sensitive. These patterns of correlation among lipids suggest a coordination of synthetic and secretory processes to create a lipidome that is reflective of metabolic status, although the tissue sources of the lipid species detected in circulation is not known. Nonetheless, the underlying regulatory mechanisms dictating the composition of the plasma lipidome appear to be altered in the metabolically impaired animals as insulin resistance develops, ahead of loss in insulin sensitivity, well in advance of IFG, and independent of differences in adiposity.

### Correlations between ceramides and adiponectin are lost early in development of insulin resistance

**Figure 6:**
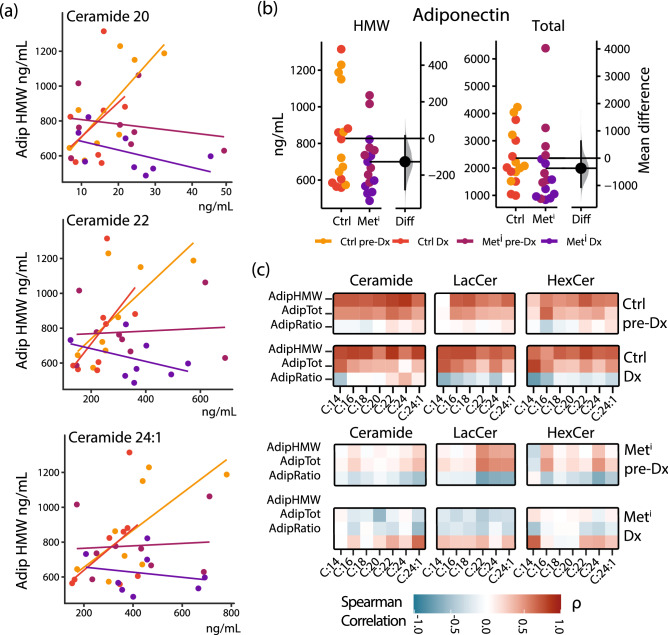
Adiponectin interactions with ceramides are dependent on metabolic health status. (**a**) Linear regressions of indicated ceramides against high molecular weight adiponectin, individual animals shown as scattered dots colored by cohort. (**b**) Estimation plots showing abundance of high molecular weight (HMW) adiponectin and total adiponectin, with mean difference and bootstrapped confidence interval between (Ctrl) and metabolically impaired (Met^i^) animals pre-diagnosis (pre-Dx) and at time of diagnosis (Dx). (**c**) Heatmap displaying Spearman’s rank correlation between high molecular weight adiponectin (AdipHMW) adiponectin, total adiponectin (AdipTot), and the ratio of HMW/Total (AdipRatio), against ceramides, hexosylceramides (HexCer) and lactosylceramides (LacCer) of varying chain lengths. Regressions colored from − 1 to + 1 according to legend.

Adiponectin is an adipose-derived peptide hormone that activates lipid utilization and mitochondrial oxidative pathways in target tissues^22,23^. Adiponectin has been linked to metabolic dysfunction associated with obesity^24,25^ and aging^26,27^, and has been implicated in the mechanisms of delayed aging by caloric restriction^28,29^. Adiponectin circulates as a multimer and the high molecular weight (HMW) isoform has been specifically linked to insulin sensitivity^30^. In adiponectin responsive tissues, adiponectin receptors activate ceramidase, tying intracellular ceramide processing directly to intracellular bioenergetic sensory mechanisms^31^. At time of diagnosis, the three species of ceramides that were significantly increased with insulin resistance (20, 22, and 24:1) each showed strong correlations with high molecular weight adiponectin in healthy animals (Fig. 6a), despite considerable differences in their abundance. This contrasts with the impaired animals, where ceramides did not correlate with high molecular weight adiponectin, even at the earlier time point when the pre-impaired animals were clinically healthy. Differences in correlations between healthy and impaired are not explained by overt differences in adiponectin levels (Fig. 6b). Comparing cohort differences shows modest and not significant reductions in HMW and total Adiponectin. A consistent pattern of correlations between either total or high molecular weight isoforms of adiponectin and each of the sphingolipid species (ceramide, lactosylceramide, hexosylceramide) was detected in the healthy animals at both time points but was absent in the impaired animals at both time points (Fig. 6c). We previously reported that, high molecular weight adiponectin was featured in a predictive model derived from lipoprotein, lipid, and adipokine profiles^8^. These new data potentially link circulating ceramides and their glycosphingolipid derivatives to HMW adiponectin, and thereby to adipose tissue function.

## Discussion

A well-established association exists between metabolic disorders and increased abundance of circulating and tissue resident lipids^32^. The signaling properties of many lipids, which allow for modulation of metabolic homeostasis in the healthy metabolic state, are thought to instigate a disruptive cascade during metabolic impairment. Excessive and ectopic lipid accumulation that occurs with obesity compromises normal metabolic regulatory mechanisms, impacting function at the tissue level and further contributing to homeostatic imbalance^23,33,34^. The current study involved groups of age, weight, and adiposity matched animals, and was designed specifically to exclude differences in adiposity as a contributing factor in the development of insulin resistance. The focus was to identify lipid-related changes associated with metabolic disease progression, and to determine if changes occurring early in the development of insulin resistance might identify disease risk independent of adiposity and in advance of IFG. A limitation of the study is that there were many more observations (clinical measures quantified, lipid species detected) than there were animals, even taking into consideration the two time point design. Throughout we report unadjusted p values and favor patterns of difference identified via lasso and regression matrices as a means to uncover the involvement of lipids in spontaneous of insulin resistance development.

Metabolomic analysis has proved successful in identifying changes in circulating polar or soluble metabolites and derivatives in persons with metabolic syndrome and type 2 diabetes, but in most cases the impaired cohorts already exhibit IFG if not frank diabetes^35^. In lipidomic studies of pre-diabetes, ceramides of C16:0, C18:0, C20:0, and C24:0 carbon chain lengths were positively associated with fasting insulin glucose levels and IFG while the association was reversed for sphingomyelins of C16:0, C18:0, 20:0, and C24:0 carbon chain length^11,36^, although the latter seemed to be driven by differences in BMI among the cohort. Independent studies have also reported increases in dihydroceramides in metabolically compromised individuals^37^. The findings reported here align with those of human studies, but differ in the timing of disease progression (Fig. 1a). Data shown here indicate that changes in abundance of circulating ceramides occur in advance of development of IFG, and because the cohorts were matched for bodyweight and adiposity that was unchanged over the study period these changes cannot be attributed to differences in body composition.

Recent reviews emphasize the potential importance of ceramides as biomarkers of metabolic dysfunction^38,39^. Numerous publications have noted increased ceramide content in liver, muscle, adipose tissue, and plasma in genetically or diet-induced insulin resistance in mice^40–42^. Studies of laboratory rodents have the advantage of matched dietary intake and a tightly controlled environment; however, issues of genetic homogeneity and the physiological relevance of high fat diet feeding are a concern in translating findings to human populations. In the current study, genetically heterogeneous monkeys were maintained in a controlled environment for their entire adult lifespan, ate the same chow diet, and were monitored for glucoregulatory function every 6 months. In this way the onset of insulin resistance was captured within a small time-window. A prior cross-sectional study detected elevated plasma ceramide levels in monkeys fed a high fat diet when compared to controls (Brozinick et al*.*, 2013). In the current study, the development of insulin resistance was spontaneous rather than diet induced. The longitudinal design accommodates the natural variance among genetically heterogeneous animals while taking advantage of the uniformity of husbandry and diet, and high frequency of clinically relevant physiological and metabolic assessments that allowed insulin resistance onset to be captured. Another advantage is animals were equivalent in adiposity and glycemic status despite divergence in insulin sensitivity status, allowing for the early stages in loss of insulin sensitivity to be interrogated in the absence of differences in these clinically associated factors. With this highly controlled study design we confirm that circulating ceramides and de novo precursors are higher in abundance in metabolically impaired animals (Fig. 2b,c), while conversely simple glycosphingolipids hexosylceramides and lactosylceramides are less abundant in metabolically impaired animals, demonstrating that sphingolipids are perturbed in concert with metabolic dysfunction (Fig. 3c,d). Although not measured in this study, it would be interesting to determine whether 1-deoxysphingolipids, previously linked to type 2 diabetes in humans^43^, are produced in the early stages of spontaneous insulin resistance development in nonhuman primates.

Correlational analysis between biometric health indices of body weight, adiposity, glucoregulatory parameters, and circulating adipokines, against lipid species DAG and NEFA revealed significant differences between impaired and healthy groups, not only at diagnosis but 2 years prior when the animals were clinically indistinguishable (Fig. 1c). These data suggest a potential change in how circulating lipid species relate to adipose function during progression to insulin resistance. For ceramides the interactions within lipid class were diminished even before loss of insulin sensitivity was detected clinically, and interactions among DAG and to a lesser extent NEFA were altered (Fig. 4a). Sphingomyelins are more abundant in circulation than any of the other species quantified in this study, and while there are strong interactions among sphingomyelins, these do not appear to extend to other lipid species. Although they were of lower abundance in the impaired, the considerable variance was the more striking feature (Fig. 3e). Feedback loops regulating lipid synthesis are influenced by factors in the insulin signaling pathway suggesting that lipid metabolic pathways may be particularly sensitive to changes in insulin effectiveness^44^. Regression analysis across lipid species (Fig. 5) suggests that lipid metabolism in general is highly responsive to insulin sensitivity status. The absence of correlations between ceramides and HMW adiponectin in impaired animals indicate connections between circulating lipids and adipose tissue function may be altered during the development of metabolic impairment (Fig. 6c). This study only investigated circulating lipid species; however, ceramides and DAG each have been identified as having intracellular signaling capacity^41^ and were among the most responsive to future loss of insulin sensitivity. As the tissue sources of circulating ceramides, glycoceramides, and sphingomyelins have yet to be identified, it is difficult to relate changes in circulation with specific intracellular lipid synthetic processes. The changes in lipid distribution identified here do not rule out the possibility that these species might contribute to development of metabolic impairment although that remains speculation at this stage.

In summary, data reported here indicate that circulating plasma sphingolipid species are altered early in the development of insulin resistance, independent of changes in adiposity, and prior to loss of euglycemia. The interactions among circulating sphingolipids and among other insulin responsive biomolecules such DAG and HMW adiponectin that are apparent in healthy animals are lost in the impaired animals even in advance of insulin resistance onset. We propose that lipid profiling would be a valuable avenue for identification of early warning biomarkers of insulin resistance, and for studies focused on the underlying biology of metabolic impairment.

## Supplementary Information

Supplementary Information 1.

Supplementary Information 2.

Supplementary Information 3.

## Data Availability

Data will be made available upon request to Dr. Rozalyn Anderson.

## Abbreviations

DAG: Diacylglycerol
ER: Endoplasmic reticulum
NEFA: Nonesterified fatty acid
FSIGT: Frequently sampled intravenous glucose tolerance test
HMW: High molecular weight
IFG: Impaired fasting glucose
IQR: Interquartile range
OR: Odds ratio
Si: Insulin sensitivity index
SM: Sphingomyelin
TAG: Triacylglycerol
