# Supplementary material for: Ceramides are early responders in metabolic syndrome development in rhesus monkeys
Source: Sci Rep. 2022 Jun 15;12:9960. doi: 10.1038/s41598-022-14083-3 (PMC9200850; doi:10.1038/s41598-022-14083-3)
Supplement: Supplementary file 3 — Supplementary Information 3. [file 41598_2022_14083_MOESM3_ESM.docx]

**Supplemental Figure Legends:**

**Supplement Figure 1**. Pathway depicting main sphingolipid synthesis pathways including *de novo*, salvage pathway, sphingomyelin, and downstream complex glycosphingolipid formation.
